# A case of delayed acute intestinal pseudo-obstruction after MELAS crisis in mitochondrial encephalomyopathy with lactic acidosis and stroke-like episodes

**DOI:** 10.3389/fmed.2026.1760790

**Published:** 2026-01-15

**Authors:** Qinggen Liu, Lina Yang, Pengcheng Liu, Xipeng Wang, Kaiyue Peng, Qi Liu, Kun Zhang

**Affiliations:** 1 Department of Gastrointestinal Surgery, Jinan Zhangqiu District People’s Hospital, Jinan, China; 2 Department of Endocrinology, Jinan Zhangqiu District People’s Hospital, Jinan, China; 3 Department of Neurology, Jinan Zhangqiu District People’s Hospital, Jinan, China; 4 Department of Thyroid and Breast Surgery, Jinan Zhangqiu District People’s Hospital, Jinan, China

**Keywords:** acute intestinal pseudo-obstruction, case report, jejunal decompression, MELAS, mitochondrial encephalomyopathy

## Abstract

**Introduction:**

Mitochondrial encephalomyopathy with lactic acidosis and stroke-like episodes (MELAS) is a rare mitochondrial disorder primarily presenting with neurological manifestations, but it may also lead to gastrointestinal complications such as intestinal pseudo-obstruction, which can significantly complicate clinical management.

**Case presentation:**

We report a 24-year-old woman with genetically confirmed MELAS (mtDNA A3243G mutation and TRPM3 c.2878G > T heterozygous variant) who was admitted with acute confusion and generalized convulsive seizures. Neuroimaging revealed multiple low-density lesions on CT and abnormal signals in the bilateral occipital lobes and basal ganglia on MRI. Although her neurological symptoms stabilized after emergency treatment, she subsequently developed recurrent episodes of intestinal obstruction characterized by vomiting and decreased bowel sounds. Conservative measures were unsuccessful, and jejunal decompression was performed, resulting in significant improvement in bowel function and successful transition to normal oral intake.

**Conclusion:**

This case illustrates delayed acute intestinal pseudo-obstruction (AIPO) as an uncommon but clinically important complication of MELAS, and shows that timely jejunal decompression can be an effective therapeutic intervention in selected patients.

## Introduction

Mitochondrial encephalomyopathy with lactic acidosis and stroke-like episodes (MELAS) is a rare genetic disorder caused by mutations in mitochondrial DNA, resulting in multisystemic dysfunction (1). It mainly presents with neurological symptoms like stroke-like episodes, seizures, and cognitive decline (2). Rarely, MELAS can also cause complications such as intestinal pseudo-obstruction, which can complicate treatment (3). We report a case of a critically ill 24-year-old woman with MELAS who experienced two episodes of intestinal obstruction during hospitalization. Initial conservative treatment was ineffective, but her condition improved after small bowel decompression.

## Case presentation

On July 7, 2025, a 24-year-old Chinese woman was urgently admitted to the hospital due to acute confusion and generalized convulsive seizures. Three months prior to admission, she had been under significant mental stress due to a debt issue. Upon admission, the patient was unconscious, with eyes closed, mouth open, and exhibiting convulsions every 4–5 min, accompanied by urinary incontinence and vomiting. There was no fever or tongue biting. Her past medical history was unremarkable, with no similar episodes or history of systemic diseases, cerebrovascular diseases, or chronic conditions. She had no history of adverse drug use, toxins, smoking, or alcohol consumption, and no family history of similar symptoms. The patient appeared emaciated, weighing 35 kg (BMI 13.67 kg/m^2^), with marked generalized muscle wasting and reduced muscle bulk. Her heart rate was 130 bpm, and she was uncooperative with the examination. Her consciousness was at a shallow coma level. Cranial nerve examination could not be performed, and muscle tone was normal. The strength and coordination tests could not be completed due to non-cooperation. Abdominal reflexes and other superficial reflexes were normal. Deep reflexes, including biceps, triceps, brachioradialis, and Achilles tendon reflexes, were normal. All pathological reflexes, including Babinski, were negative. Meningeal irritation signs were absent.

**Figure 1:**
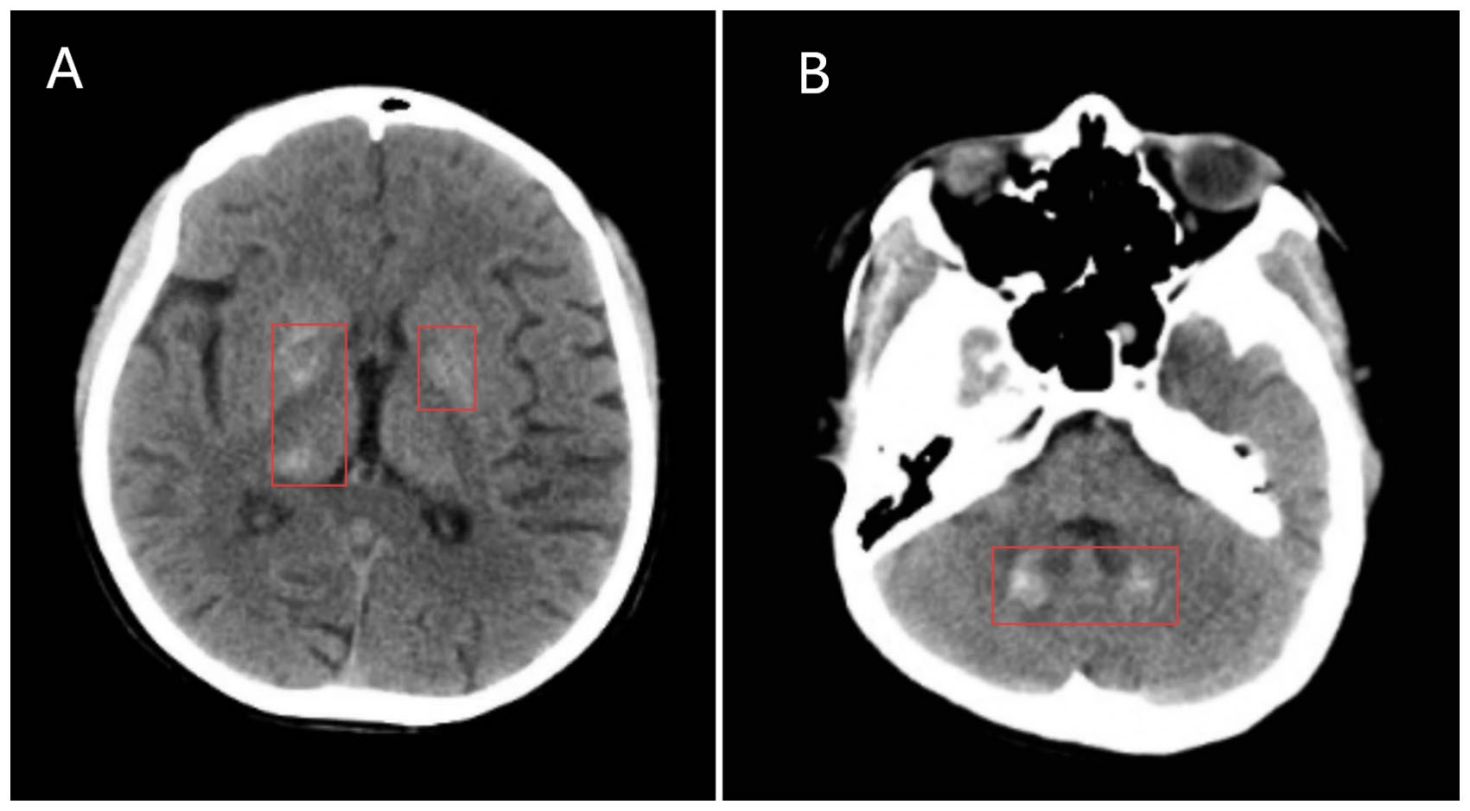
Head CT. **(A)** High-density lesions in the bilateral basal ganglia (red mark). **(B)** High-density lesions in the cerebellar dentate nuclei (red mark).

**Figure 2:**
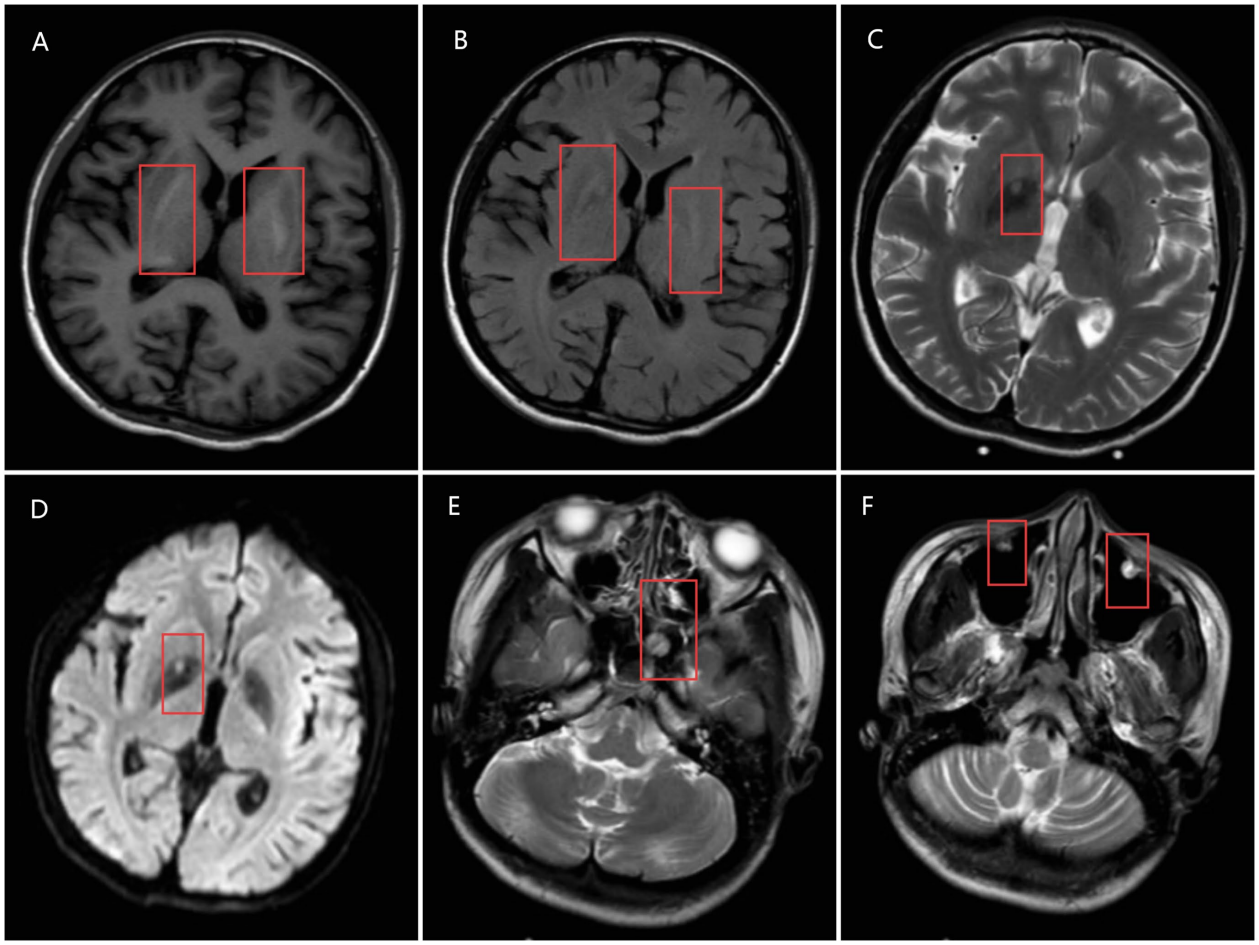
Head MRI. **(A)** T1-weighted image showing hyperintense lesions in the bilateral basal ganglia and thalamus (red mark). **(B)** T2-FLAIR image showing hyperintense lesions in the bilateral basal ganglia and thalamus (red mark). **(C)** Dot-like abnormal signal in the right putamen on T2-weighted images (red mark). **(D)** ADC map showing restricted diffusion in the right putamen (red mark). **(E)** Rounded hyperintense focus in the sphenoid sinus on T2-weighted images (red mark). **(F)** Hyperintense focus in the bilateral maxillary sinuses on T2-weighted images (red mark).

Immediate fluid resuscitation and sedation were administered, with further diagnostic tests revealing the following: lactate 7.5 mmol/L (normal range 0.5–1.8 mmol/L), white blood cell count 16.46 × 10^9^/L (elevated), neutrophil percentage 88.6%, *β*-hydroxybutyrate 0.338 mmol/L (0–0.3), HbA1c 15%, procalcitonin (PCT) 4.4 ng/mL (normal range 0–0.05 ng/mL), high-sensitivity troponin (HS-TnT) 9.46 pg./mL (normal range 0–14 pg./mL), CKMB-M 13.5 ng/mL (normal range 0.1–4.94 ng/mL), myoglobin 139 ng/mL (normal range 25–58 ng/mL), proBNP 377 pg./mL (normal range 0–125 pg./mL). Blood chemistry showed abnormal liver and heart function: AST 64 U/L (normal range 13–35 U/L), ALT 100 U/L (normal range 7–40 U/L). The patient had signs of abnormal cardiac and liver function. Head Computed Tomography (CT) revealed multiple slightly low-density lesions in the left temporal, parietal, and occipital lobes, as well as the right occipital lobe, with shallow local sulcal and fissural flattening and widening of the remaining sulci. Symmetrical, slightly high-density areas were observed in the bilateral basal ganglia, thalamus, and cerebellar dentate nuclei, with unclear borders and CT values ranging from 50 to 70 HU. No midline shift was detected. These findings were consistent with metabolic encephalopathy (Figure 1). Chest CT showed bronchial wall thickening, partial bronchial narrowing, and multiple patchy and nodular high-density areas around the bronchi, with indistinct borders, as well as partial lung consolidation. No significant lymph node enlargement was seen in the mediastinum, and no pleural effusion was present. These imaging features were suggestive of bronchopneumonia with partial lung consolidation. Head Magnetic Resonance Imaging (MRI) demonstrated more pronounced abnormal signals in the left temporal, parietal, and occipital lobes, with some areas showing unclear signals. Diffusion-weighted imaging (DWI) revealed restricted diffusion in both occipital lobes, along with small, patchy, slightly low T1 and slightly high T2 signals in the bilateral basal ganglia and thalamus. A small, dot-like abnormal signal was observed in the right putamen on both T1 and long T2 images. The ventricular system was mildly dilated, and the brain sulci and fissures were both widened and deepened. No significant midline shift was noted. These MRI findings considered the diagnosis of metabolic encephalopathy (Figure 2).

The patient was immediately transferred to the ICU, where she was intubated and placed on a ventilator. Sedation and analgesia with propofol, levorphanol, and remifentanil were administered. Respiratory parameters were closely monitored and adjusted. At night, her respiratory rate fluctuated between 10 and 20 breaths per minute, heart rate ranged from 110 to 125 bpm, and blood pressure was 94/55 mmHg. Her oxygen saturation ranged from 95 to 99%. Since she was unable to tolerate oral feeding, parenteral nutrition was started. The patient was diagnosed with status epilepticus, MELAS, pneumonia, and acute myocardial injury. She received appropriate treatment including levetiracetam 1,500 mg BID, lamotrigine 25 mg QD for epilepsy, dehydration therapy to reduce cerebral edema, and supplements like arginine, idebenone, coenzyme Q10, and L-carnitine to improve energy metabolism. Antibiotics, including moxifloxacin, were given for infection management, and phosphocreatine sodium was used to support myocardial function. After intensive organ support and therapy, her condition improved, and on July 14, 2025, her endotracheal tube was removed. Her lactate level decreased to 3.2 mmol/L.

**Figure 3:**
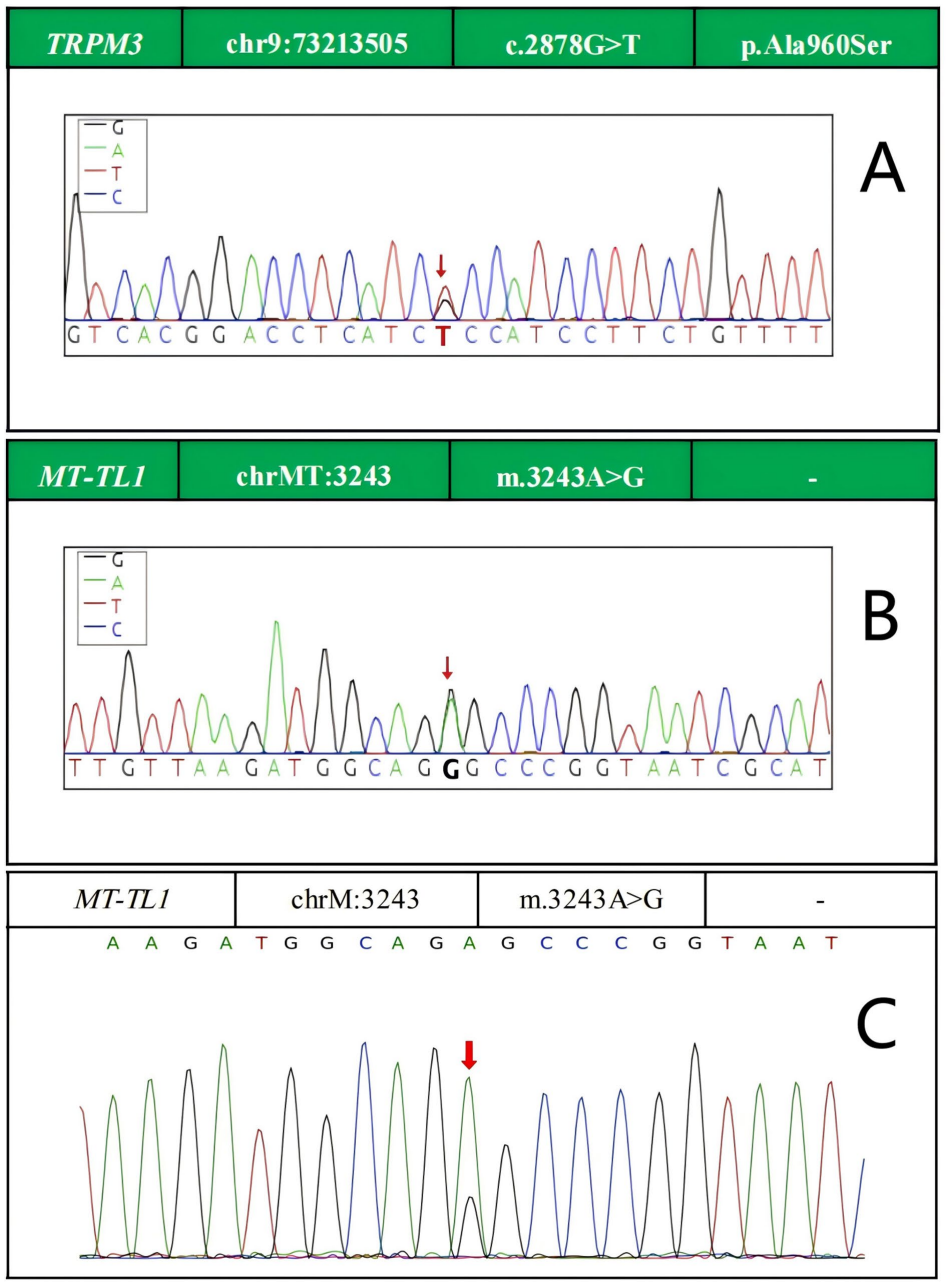
Genetic testing. **(A)** *TRPM3* gene mutation (red arrow). **(B)** *MT-TL1* gene mutation (red arrow). **(C)** Sister’s genetic test showing *MT-TL1* gene mutation (red arrow).

On July 14, 2025, genetic testing confirmed the diagnosis of MELAS syndrome. Genomic DNA was extracted from EDTA-anticoagulated whole blood, and sequencing was performed using target region capture with high-throughput sequencing. The results showed: (1) a heterozygous variant in the TRPM3 gene, c.2878G > T (chr9:73213505), p.Ala960Ser (Figure 3A), with a sequencing depth of 100/92 and a variant allele fraction of 0.48; (2) a pathogenic mitochondrial variant in MT-TL1, m.3243A > G (NC_012920.1) (chrMT:3243, rs199474657) (Figure 3B), with a variant read depth/total depth of 4307/7023, corresponding to an estimated heteroplasmy level of 61.3% in whole blood. In addition, genetic testing in her sister also identified the MT-TL1 m.3243A > G variant (Figure 3C).

**Figure 4:**
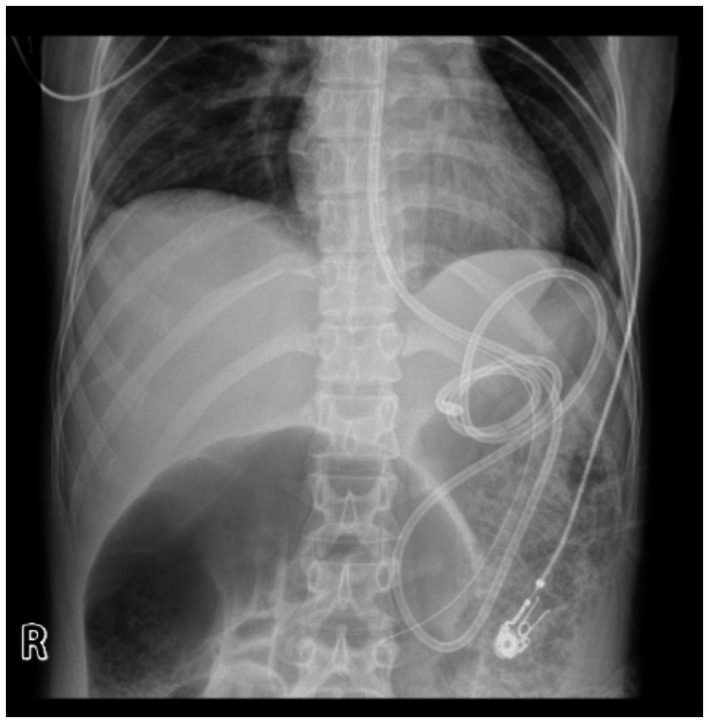
Abdominal X-ray. The small bowel gas accumulation and bowel dilation during gastrointestinal tube placement.

**Figure 5:**
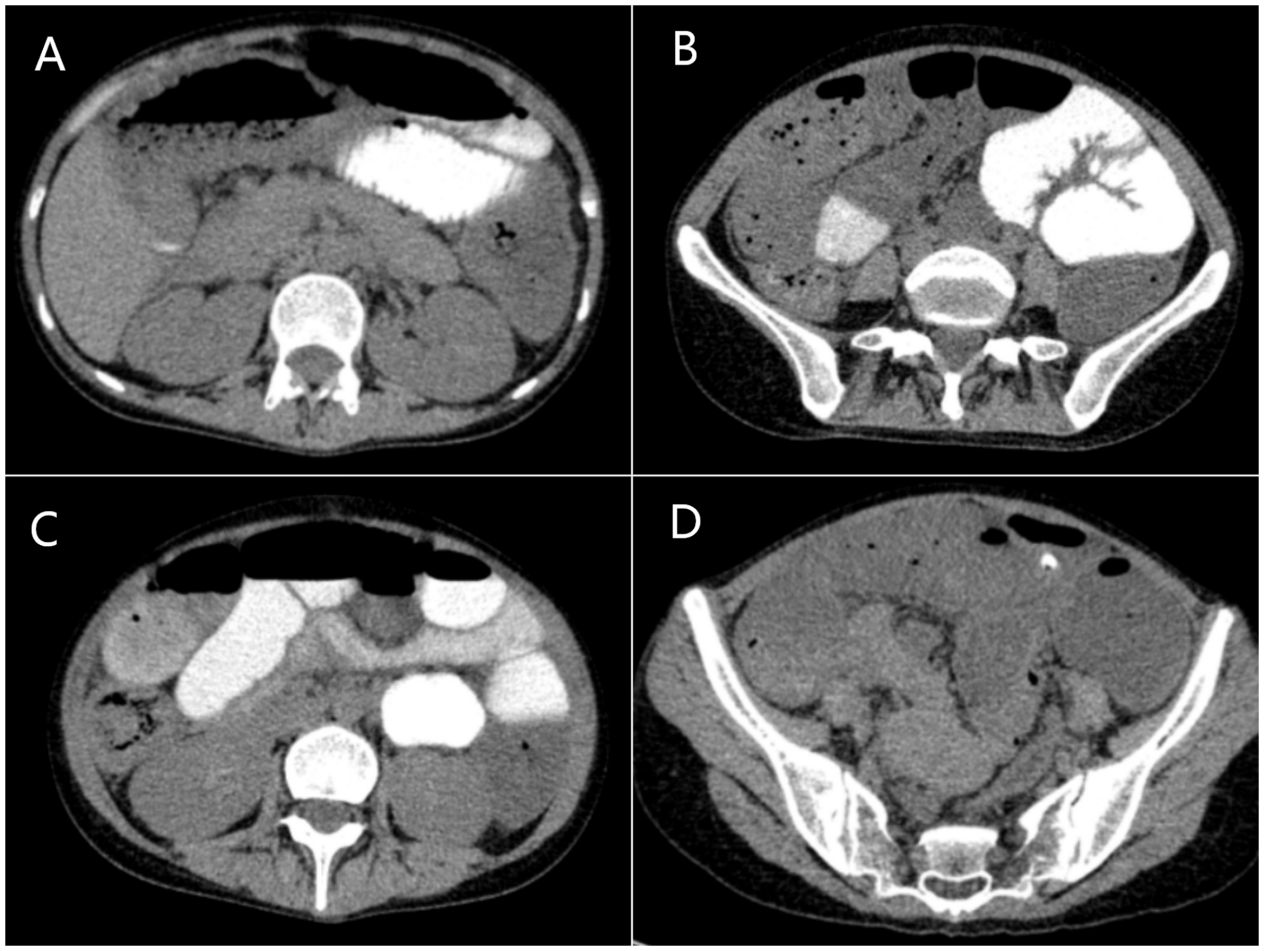
The abdominal CT during the second episode of bowel obstruction. **(A–D)** Axial scans showing gas accumulation and bowel dilation in the small intestine.

**Figure 6:**
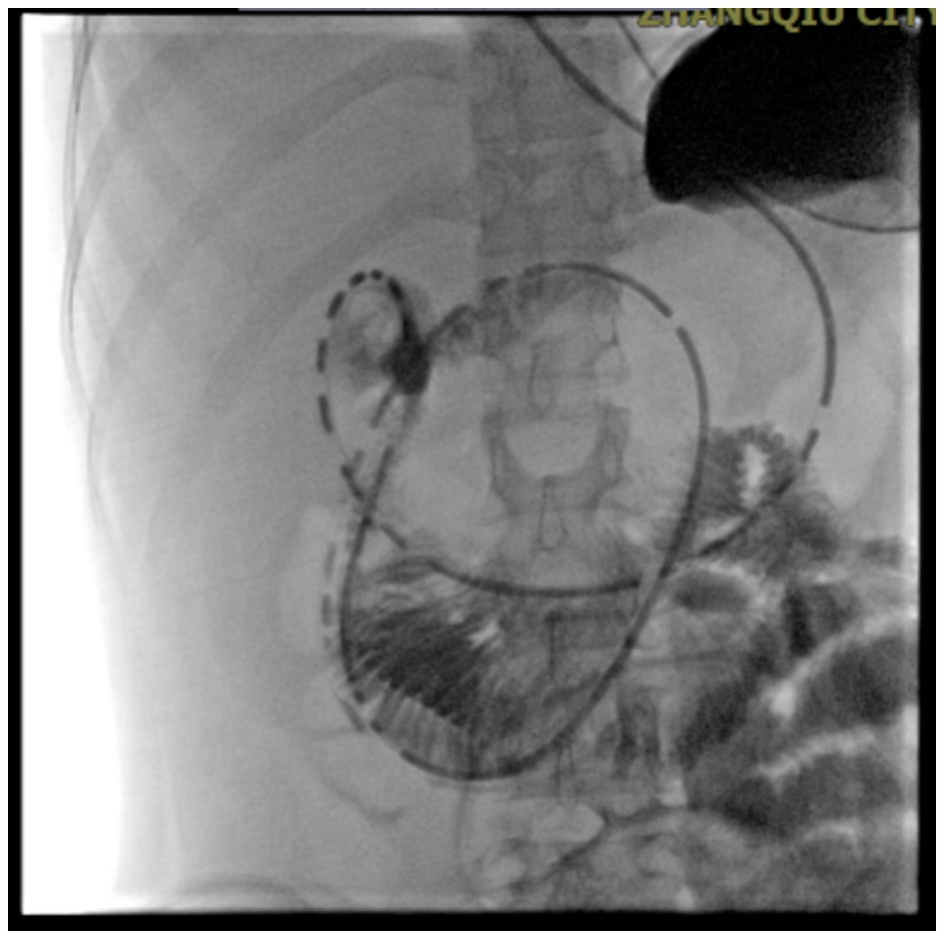
Small bowel decompression. Small-bowel decompression tube inserted under interventional guidance.

Gastrointestinal obstruction: On July 14, 2025, the patient developed the first episode of bowel obstruction, with severe abdominal distension and reduced bowel sounds at 1 per minute. An abdominal X-ray in the upright position showed gas accumulation and bowel dilation, observed during gastrointestinal tube placement (Figure 4). CT scan revealed air-fluid levels in part of the small bowel. Abdominopelvic CT showed diffuse small bowel dilatation with air–fluid levels without an identifiable transition point or mechanical obstructing lesion, and there was no imaging evidence of volvulus, hernia, or mass. After gastrointestinal decompression, enteral nutrition was administered, and the patient’s condition improved. She gradually resumed a liquid diet and then a normal diet, while continuing treatment for MELAS. Despite this, the patient experienced a second episode of bowel obstruction from August 20 to August 23, 2025, with progressive abdominal distension, absence of gas or stool passage, abdominal pain, nausea, and vomiting. Repeat CT again demonstrated marked bowel dilatation with air–fluid levels without a definite transition point or obstructing lesion, supporting the diagnosis of acute intestinal pseudo-obstruction rather than mechanical obstruction. (Figure 5). The patient was unable to take oral anticonvulsant medications, so diazepam was administered intravenously via a micro-pump. A nasogastric tube was inserted for decompression, and a small-bowel decompression tube was placed under interventional guidance (Figure 6). After 12 days of treatment with enema, enteral and parenteral nutrition, the patient’s bowel function gradually recovered. Bowel sounds returned to normal, and she was successfully discharged after transitioning to a normal diet. A follow-up phone call 16 days post-discharge revealed no adverse events.

## Discussion

Mitochondrial encephalomyopathy (ME) is a genetic metabolic disorder caused by mutations in the mitochondrial and/or nuclear DNA, leading to structural and/or functional mitochondrial dysfunction, which results in impaired cellular energy supply in multiple organs. The disease commonly manifests with symptoms affecting the brain and muscles (1, 4). MELAS is the most common type of mitochondrial encephalomyopathy. It is predominantly inherited maternally, with the peak onset age between 10 and 30 years. Onset after the age of 40 is rare, and those presenting with stroke-like episodes after this age are generally referred to as late-onset MELAS (4, 5). The most common genetic mutation in MELAS patients is the mtDNA A3243G point mutation. Stroke-like episodes are the most common initial clinical manifestation, accounting for approximately 70.53% of cases (6). Due to mitochondrial dysfunction, ATP production is reduced, leading to increased oxidative stress, lactate accumulation, and subsequent clinical symptoms such as stroke-like episodes, epilepsy, cognitive and psychiatric disorders, exercise intolerance, sensorineural hearing loss, peripheral neuropathy, gastrointestinal dysfunction, cardiomyopathy, arrhythmia, endocrine abnormalities, hirsutism, and skin abnormalities (4). Gastrointestinal dysmotility is not uncommon in MELAS; in a recent retrospective cohort, gastric dysmotility was reported in 5 of 42 (11.9%) patients meeting MELAS criteria (7). In this case, the patient’s first symptoms were stroke-like episodes accompanied by hyperlactatemia. Brain MRI showed T2 FLAIR hyperintensities in the bilateral basal ganglia, thalamus, and right putamen. Genetic testing confirmed the mtDNA A3243G mutation and the c.2878G > T heterozygous variant, leading to a diagnosis of MELAS. The mtDNA A3243G mutation is widely recognized as a key pathogenic variant in MELAS. In addition, TRPM3 is a cation channel sensitive to temperature and neurosteroids, expressed in both neuronal and non-neuronal cells. Recent studies have identified rare gain-of-function mutations in TRPM3, which lead to a spectrum of neurodevelopmental disorders, including intellectual disability, epilepsy, and cerebellar ataxia (8). Although its direct role in MELAS is not yet fully understood, it may contribute to the clinical symptoms, particularly in relation to seizures, as observed in this case. This case suggests that TRPM3 could be a potential mechanism, offering new insights to further elucidate the underlying pathology.

Due to the incomplete understanding of the pathogenesis and causative genes of mitochondrial encephalomyopathy, there is currently no specific treatment for this condition. The main approach is symptomatic treatment and supportive care, as no drug with proven efficacy has been developed so far. The progression and treatment prognosis vary depending on the subtype of the disease. Coenzyme Q10, L-carnitine, and creatine may improve mitochondrial function and muscle strength, as well as reduce blood lactate, benefiting some patients (9, 10). Furthermore, comprehensive supportive therapy, including circulatory support, metabolic regulation, and organ function protection through multidisciplinary teamwork, is essential (11). Gene therapy remains the most fundamental and promising cure, with basic strategies including (12): reducing the ratio of mutated mtDNA to wild-type mtDNA, introducing homologous genes, using heterologous and misdirected expression, and employing restriction endonucleases to repair mutated mtDNA. Specific methods include: RNA gene insertion into mitochondria, nucleic acid product insertion, and peptide insertion into mitochondria. The five commonly used techniques include: constructing mtDNA plasmids, compensatory expression of exogenous genes in mitochondria, cell fusion and mitochondrial transfer technologies, compensatory expression of mitochondrial genes, and removal of defective mitochondrial genomes (13, 14). However, these techniques are still in the research stage and have not been widely used in clinical practice, as there is no large-scale clinical data to support their efficacy (12, 15).

In addition to stroke-like episodes, acute intestinal pseudo-obstruction (AIPO) is an uncommon manifestation of mitochondrial encephalomyopathy. Intestinal pseudo-obstruction, first described by Dudley et al. (16) in 1958, is a disabling motility disorder characterized by severe symptoms and signs of intestinal obstruction, such as abdominal pain, distention, nausea, and vomiting, along with radiographic evidence of bowel dilation in the absence of any mechanical obstruction. While typically regarded as a small-bowel motility disorder, pseudo-obstruction can occur anywhere in the gastrointestinal tract. It can present as either an acute condition, such as Ogilvie syndrome, or a chronic, remitting, or persistent disorder. In cases of small bowel obstruction, the inability to administer oral medications complicates treatment. Seessle et al. (17) reported a case of a 26-year-old MELAS patient who developed acute paralytic ileus. Conservative treatments proved ineffective, but high-caloric parenteral nutrition through a central venous catheter resulted in rapid clinical improvement. Similarly, Nakae et al. (18) described a MELAS patient with chronic intestinal pseudo-obstruction (CIPO) who showed improvement after receiving distigmine bromide, which may work by targeting acetylcholine receptors on the interstitial cells of Cajal to enhance bowel motility. Kawano et al. (19) also reported a 51-year-old MELAS patient with CIPO whose symptoms, including nausea, vomiting, and abdominal distension, gradually improved following treatment with acotiamide. The mechanisms of intestinal dysmotility in MELAS may be linked to mitochondrial dysfunction affecting smooth muscle cells and the enteric nervous system, leading to impaired motility. Reduced mitochondrial ATP production and lactate accumulation, common in MELAS, may weaken the contractility of intestinal smooth muscle, contributing to intestinal pseudo-obstruction. Mitochondrial dysfunction may also affect the enteric nervous system, disrupting the regulation of gut motility (14). Our patient experienced two episodes of intestinal obstruction. The first episode occurred during a period of critical illness, with the patient being exposed to sedatives and systemic stress, which may have contributed to the development of paralytic ileus. To exclude mechanical obstruction, an abdominopelvic CT scan was performed, with careful evaluation for a transition point. The results showed diffuse small bowel dilatation with air–fluid levels but no obstructing lesion, no definite transition point, and no imaging evidence of volvulus, hernia, or mass. A surgical consultation was obtained, and it was determined that mechanical obstruction was unlikely. The patient was therefore managed as having acute intestinal pseudo-obstruction (AIPO). Despite initial conservative treatments, including total parenteral nutrition and gastrointestinal decompression, the symptoms showed limited improvement. After the neurological condition stabilized, and with the discontinuation of sedatives and analgesics, the second episode of bowel obstruction occurred, reinforcing that the condition was unlikely to be related to ICU-associated ileus. This recurrence of non-mechanical intestinal dysmotility, in the absence of persistent electrolyte imbalances or other ICU-related factors, further supports the diagnosis of MELAS-related intestinal involvement rather than a transient ICU-associated ileus. After jejunal decompression tube insertion, the symptoms improved. Therefore, early insertion of a jejunal decompression tube can be an effective treatment option when a MELAS patient presents with acute intestinal obstruction.

In conclusion, intestinal obstruction is a rare complication of MELAS. Jejunal decompression helped resolve the patient’s symptoms, underscoring the need for prompt treatment in AIPO cases in MELAS patients.

## Data Availability

The original contributions presented in the study are included in the article/supplementary material, further inquiries can be directed to the corresponding author.
